# Self-assessment and learning motivation in emergency point-of-care ultrasound: an online pilot investigation in German physicians

**DOI:** 10.1186/s12873-024-01154-z

**Published:** 2024-12-18

**Authors:** Joachim Bansbach, Michael Bentele, Matthias Bollinger, Stefanie Bentele, Ronny Langenhan, Bianka Gerber, Milena Trifunovic-Koenig, Stefan Bushuven

**Affiliations:** 1 Department of Anesthesiology and Critical Care, Medical Center - University of Freiburg, Faculty of Medicine, University of Freiburg, Freiburg, Germany; 2 Training Center for Emergency Medicine (NOTIS e.V), Engen, Germany; 3 Institute for Anesthesiology, Intensive Care, Emergency Medicine and Pain Therapy, Hegau-Bodensee Hospital, Singen, Germany; 4 Faculty of Medical and Life Sciences, University Furtwangen, Villingen-Schwenningen, Germany; 5 Faculty of Health, School of Medicine, Witten/Herdecke University, Witten, Germany; 6 Department for Emergency Medicine, University-Hospital Augsburg, University of Augsburg, Augsburg, Germany Institute for Medical Education, University Hospital, LMU Munich, Munich, Germany; 7 Department of Orthopaedic Surgery, Hegau-Bodensee-Klinikum Singen, Virchowstrasse 10, D-78224 Singen, Germany; 8 Department of Orthopaedic Surgery, Klinikum Chemnitz gGmbH, Flemmingstraße 2, D-09116 Chemnitz, Germany; 9 Institute for Infection Control and Infection Prevention, Hegau-Jugendwerk Gailingen, Health Care Association District of Constance, Virchowstrase 10, 78224 Singen, Germany; 10 Institute for Medical Education, University Hospital, LMU Munich, Munich, Germany

**Keywords:** Overconfidence, Ultrasound, Sonography, EFAST, POCUS, Learning, Training metacognition

## Abstract

**Introduction:**

Learning motivation is essential to obtain and maintain ultrasound competencies in emergency medicine. One’s competencies herein and the need for ongoing training are best evaluated by self-assessment. This may be flawed by overconfidence effects - the belief to be better than others or better than tests reveal. This study aims to clarify the underinvestigated interaction of learning motivation and self-assessment in emergency point-of-care-ultrasound (POCUS).

**Methods:**

In this cross-sectional multicenter project, physicians assessed their own and others’ competence and learning motivation using the Situational Motivation Scale comprising intrinsic motivation, external and identified regulation, and amotivation. In addition, we presented eight ultrasound loops of different pathologies to emergency physicians of various specialties.

**Results:**

Overall, the motivation to learn was high, while self-assessment showed no significant overconfidence in POCUS. The rate of correct diagnoses based on the loops was relatively low. As a result, we did not detect overconfidence effects in participants who completed questions (*n* = 86) and tests (*n* = 56). Overplacing oneself above peers negatively correlated with intrinsic learning motivation and identified regulation and positively correlated to amotivation. Further analyses indicated that learning motivation was associated with the interactions of the physicians’ risk perception, speciality, and self-assessment.

**Discussion:**

The absence of overconfidence effects, the complexity of learning motivation and their interaction show that prior findings in other contexts may not be easily transferable to POCUS and could be highly context-sensitive. In conclusion, this study highlights high levels of learning motivation but relatively low diagnostic accuracy in POCUS, which suggests the need for ongoing education and assessment. Ensuring that physicians continue to receive objective feedback and opportunities to refine their skills is critical for maintaining high standards of care. Despite the small sample size and other limitations of the study, the results primarily served to generate hypotheses for future research on emergency ultrasound education.

**Supplementary Information:**

The online version contains supplementary material available at 10.1186/s12873-024-01154-z.

## Introduction

### Background

Point-of-Care Ultrasound (POCUS) is an emerging competency for emergency and critical care. It can used by medical teams in pre-hospital settings, emergency departments, operating rooms, labour wards, and neonatal, paediatric, and adult intensive care units to identify life-threatening pathologies rapidly. Concerning its limitations, its impact on patient safety, and the need for education and expertise [1] POCUS protocols for different professions can provide valuable information for integrated care [2], especially in shock, trauma [3, 4], peri-arrest situations, after the return of spontaneous circulation [5] and even for obstetrics [6] and pediatric care [7, 8]. However, an error in POCUS may be critical [1, 9]. Therefore, providers must receive training in using and interpreting POCUS to acquire and maintain competence for patient safety [1]. As attending training alone is no guarantee for proficiency, providers also must ensure life-long learning motivation to deepen and preserve their competencies [10]. This is not limited to POCUS [11]. Efficient learning depends on multiple factors. Motivation plays a critical role in this process and is largely determined by self-assessment, interest, values, and external factors. Self-assessment involves understanding one’s knowledge, skills, problem-solving abilities, attitudes, and behavior, which helps learners identify areas for improvement and set realistic goals [12, 13]. Interest in the subject matter can drive engagement and make the learning process more enjoyable and effective [14]. Values, or the personal relevance of the knowledge or skills to one’s life and goals, can enhance motivation [15]. External factors include supportive environments, such as encouraging teachers, peers, and access to resources, which also contribute to the motivation to learn [16]. A growing body of evidence indicates that one’s perception of their own abilities or self-confidence is the key mediating factor in achievement efforts [17–19]. However, the connection between overconfidence effects [20, 21] and the motivation to learn remains a subject of ongoing research.

Overconfidence can be observed in a wide range of everyday skills (e.g., driving a car, quitting smoking, investments, non-medical learning, gambling, and many more) [20, 22–26]. It can be divided into overplacement (relative overconfidence, believing you are better than others, also known as better than average effect), overestimation (absolute overconfidence, believing you are better than tests show), and overprecision (believing you know the truth) [20].

A related bias is the phenomenon of clinical tribalism. This “in-group bias” leads to the assumption that one`s own social group is superior to other groups, which affects team interaction and patient safety [27].

Motivation to learn can be described by the Self-determination theory [28]. This is a comprehensive psychological framework, focusing on human motivation and personality. The theory posits that individuals have three basic psychological needs: autonomy, competence, and relatedness. Autonomy refers to the need to feel in control of one’s actions and decisions. Competence involves the need to feel effective and capable in one’s activities. Relatedness is the need to feel connected to and cared for by others. When these needs are satisfied, individuals are more likely to experience intrinsic motivation, which is the drive to engage in activities for their inherent satisfaction and enjoyment. This intrinsic motivation leads to higher levels of engagement, performance, and well-being. In contrast, extrinsic motivation is driven by external rewards or punishments. However, extrinsic motivation is complex and shaped by the same factors (competence, autonomy, and relatedness). On one end of the continuum, amotivation represents a lack of drive and difficulty in fulfilling one’s needs. Moving forward, external regulation involves actions motivated by external rewards or penalties. Identified regulation is guided by personal values and goals. At the other end, intrinsic motivation is fueled by genuine interest, enjoyment, and the inherent satisfaction derived from the activity itself [29].

To date, there is limited research on the correlation between learning motivation for POCUS and overconfidence effects, which may impact motivation by hindering metacognition—the ability to reflect on one’s own thought processes [30]. However, overconfidence should be distinguished from the ‘Dunning-Kruger Effect,’ where individuals with lower competence may struggle to recognize their own limitations, leading to inflated self-assessments [31]. The Dunning-Kruger Effect is generally considered a statistical artifact [32, 33]. In contrast, overconfidence effects are genuine and can occur across various levels of competence.

Previous projects conducted by our working group have identified overplacement and clinical tribalism in various medical competencies such as hand hygiene [34, 35], basic life support [36], and management of the second victim phenomenon [37]. In these projects, we were able to identify three different types of learners [36, 38]: motivated, confident, and competent “experts”, motivated but incompetent and overconfident “recruitables”, and unmotivated, incompetent, and overconfident “unawares”. These previous studies in other medical fields indicate that the unaware group did not consider themselves incompetent, suggesting a more complex dynamic that requires further research in an underinvestigated fields of medicine to generate further hypothesis. Understanding these dynamics may be important for developing effective strategies to enhance self-awareness, motivation, and overall competency among healthcare workers.

### Objective

This study aims to assess the presence of overconfidence in POCUS in emergency medicine and its correlation with learning motivation measured by the Situational Motivation Scale (SIMS). We hypothesised that overconfidence effects are present (H1a for overplacement and H1b for overestimation) and correlate with learning motivation (H2). Furthermore, in line with previous studies, we hypothesize that physicians can be grouped into the three distinct clusters of experts (competent and motivated), recruitables (not competent but motivated), and unawares (not competent and not motivated) as shown in previous studies (H3).

## Methods

### Study design, setting and participants

We conducted a cross-sectional, anonymous online survey of approximately 500 physicians in Germany working in emergency medicine or intensive care: Participants worked in two University hospitals, four Level III hospitals, one Level I hospital or in prehospital care. The survey was conducted between October 2022 and February 2023. Participants from anaesthesiology, critical care, trauma surgery and emergency medicine were contacted via email and clinical information systems with three reminders. Data collection was carried out by the survey platform (Umfrageonline, Enuvo GmbH, Zurich, Switzerland). Participants were eligible if they were actively involved in pre-hospital or in-hospital emergency medicine or critical care.

### Variables

The online survey, which was developed and pre-tested, included demographics, items relating to motivation to learn, self-assessment of own and others’ competence in POCUS, and risk assessment. Participants were also shown eight ultrasound loops and asked for the correct diagnosis in the free-entry field.

*Learning motivation* was measured using a German translation of the SIMS that was used in the preceding work [36, 37, 39, 40] and adapted to the setting of the POCUS training. This SIMS instrument consisted of four subscales and asked participants to reflect on their feelings and attitudes when attending a typical POCUS training course. Each subscale further consists of four items: intrinsic motivation (e.g., “Because I feel good when doing this activity”), identified regulation (e.g., “Because I am doing it for my own good”), external regulation (e.g., “Because I am supposed to do it”), and amotivation (“I don’t know; I don’t see what this activity brings me”). All items are scored on an ascending 7-point Likert Scale ranging from one, “completely disagree”, to seven, “completely agree”.

*Self-assessment regarding POCUS*. We used an exploratory six-item instrument to measure POCUS for one’s own, anaesthesiologists’, and trauma surgeons’ competencies. The competencies comprised factual knowledge, psychomotor skills, attitude, problem-solving, corrective feedback and reception of feedback and were measured on a five-point rating scale, ranging from one “completely disagree” to five “completely agree”. The scales were calculated as the mean of the six items.

*Perceived risk* was measured by two ISO 31,000 conform risk assessment [41] items on the maximum possible harm to patients from an error in POCUS and how often this occurs in their workplace. Items were scored on an ascending 5-point ordinal scale. The first item measured the maximum possible harm of incorrectly performed POCUS as one “without consequence”, two “minor—without any long-lasting effect”, three “severe—prolonged hospital stay”, four “critical—with long-lasting effects”, and five “lethal”. The second item measured how often a patient was harmed in the working environment by inadequately performed POCUS as one “uncommon (once in more than three years)”, two “seldom (once every three years)”, three “occasionally (once a year)”, four “often (once every three months)”, and five “very often (once a month)”.

*Eight ultrasound loops* of clinical cases in emergency medicine in single-plane view were anonymized before extraction from the ultrasound scanner (cases are listed in Table 1 in Supplement 1). These loops were uploaded to YouTube (R) and are only accessible with the direct link. Additionally, we described eight corresponding fictional case vignettes developed by the research team and tested for content validity by 14 physicians (see supplement 1). All information in the cases was fictious, including age and gender, to protect the anonymity of the original patients. The highest possible score was 1 (or 100%), indicating an accurate diagnosis for all cases, while a score of 0 represented an incorrect diagnosis.

**Table 1:** An example of the fictious case vignettes used (all eight cases are displayed in supplement 1)

Case	Vignette	Assessment goals
1	You care for a 25-year-old male after a motorcycle accident in the emergency department. POCUS shows the video above. A: endotracheal tube, etCO 34 mmHg B: ventilated, FiO2 0.7, Bilevel 35/5 mbar, RR 20, SpO2 100% C: NIBP 70/30 mmHg, HR 141/min, ReCap time 5 s, sinustachycardia D: GCS 3, RASS − 5, sedated, Miosis E: Temperature 36,4 °C, left forearm fracture, suspected femoral fracture left, multiple bruises S: Accident on the highway 45 min ago, intubated on site with GCS 6, NIBP 170/100 mmHg, HF 44/min and anisocoria left > right A: not known M: not known P: not known L: not known E: collided with truck (80 km/h) with his motorcycle (at least 80 km/h) ABG: pending X-Ray: not conducted yet 12 lead ECG: Sinustachycardia, normal axis, no ST changes	**Correct pathology**: Recognition of free perisplenic fluid or suspected rupture of the spleen

These cases included a motorcycle accident with perisplenic fluid due to splenic laceration (#1), obstructive cardiac shock with the D-Sign in the parasternal short axis (#2), a deteriorating patient with mesenteric ischemia and portal gas bubbles in the right subcostal plane (#3), abdominal pain with POCUS to Koller’s pouch showing no pathology (#4), a right-sided haemothorax (#5), a patient in cardiogenic shock with POCUS showing a parasternal long axis view with cardiac tamponade (#6), a patient with desaturation after central venous line placement showing the lung in M-mode without pathology (#7), and a geriatric patient with sepsis in the emergency room with pneumonia and POCUS signs of atelectasis and pleural effusion (#8). The answer was correct if the primary pathology was correctly identified. The loops can be accessed via the URLs provided in Supplement 2. The developed and pretested questionnaire is presented in Supplement 2 in an English version translated by the authors.

Relative Self-Assessment was calculated as the relative ratio of the self-assessment of one’s own and the competencies of anaesthesiologists or trauma surgeons. We were unable to calculate the Relative Self-Assessment for internal medicine physicians or pediatricians, as we did not include items that allowed them to assess their competencies in relation to their peers concerning POCUS. This ratio may be greater than or less than one, indicating whether individuals rate their own abilities higher or lower compared to their colleagues, reflecting not just overplacement but also instances where one may appreciate colleagues’ skills more than their own.

Self-evaluation to accuracy ratio was measured as the relative ratio between the self-assessment of knowledge and attitude regarding POCUS and the proportion of correctly identified pathologies of the eight ultrasound loops. We included the participants who responded to at least two out of eight cases ultrasound cases. The decision to use a minimum of two cases was driven by the small sample size, which constrained our ability to apply stricter inclusion criteria. Setting a higher threshold could have excluded too many participants, reducing the statistical power of the analysis and potentially leading to biased results. By allowing participants with at least two completed cases, we aimed to balance the need for meaningful data while retaining as many participants as possible for the analysis. Since the two variables were measured by different scales (mean of self-assessment of knowledge and attitude regarding POCUS from 1 to 5 and the proportion of correctly identified pathologies from 0 to 1), we z-standardized and added a constant of 100 to both variables to have a positive scaling on both sides of the fraction.

### Statistical methods

SPSS 29 (IBM) was used for statistical calculations. To test for the presence of overplacement (H1a), we performed the t-test for dependent samples comparing one’s own and the competencies of colleagues of the same specialty.

The presence of overestimation (H1b) was tested using the t-test for dependent samples to compare the z-transformed mean of the self-assessment of knowledge and attitudes regarding POCUS and performance on the ultrasound loops, to which we added the value of 100 after transformation.

We assessed linear correlations between Relative Self-Assessment, Self-evaluation to accuracy ratio and four dimensions of learning motivation (hypothesis 2) using Pearson’s product-moment correlation matrix.

Pearson’s correlations and paired t-tests were performed using bias-corrected and accelerated (BCa) bootstrapping method at 95% confidence intervals (BCa based on 1,000 samples).

To test H3 (the presence of three groups), a 2-step cluster analysis was performed. We included learning motivation, self-assessment regarding POCUS, the proportion of correctly identified ultrasound pathologies, and the risk to the patient after an incorrectly POCUS diagnosis as a categorical variable. We used the log-likelihood measure of distance and performed the analyses twice, choosing the Bayes Information Criterion (BIC) and the Akaike Information Criterion (AIC), respectively. Following the two-step analysis, we reviewed the mean Silhouette coefficient of cohesion and separation for the proposed cluster solution to evaluate the appropriateness of the result (values above 0.25 are considered satisfactory, and values above 0.45 are considered appropriate).

### Other analysis

Further, we conducted a post hoc analysis to examine the impact of risk perception on learning motivation and its correlation with specialty and self-assessment of competencies regarding POCUS, given the varying patterns of correlations observed across specialties. We hypothesized that specialty correlates with risk perception and explored how the interaction between specialty and risk perception, moderated by self-assessment, correlates to learning motivation. This post hoc analysis serves primarily for hypothesis generation and suggests directions for future research.

**Fig. 1:**
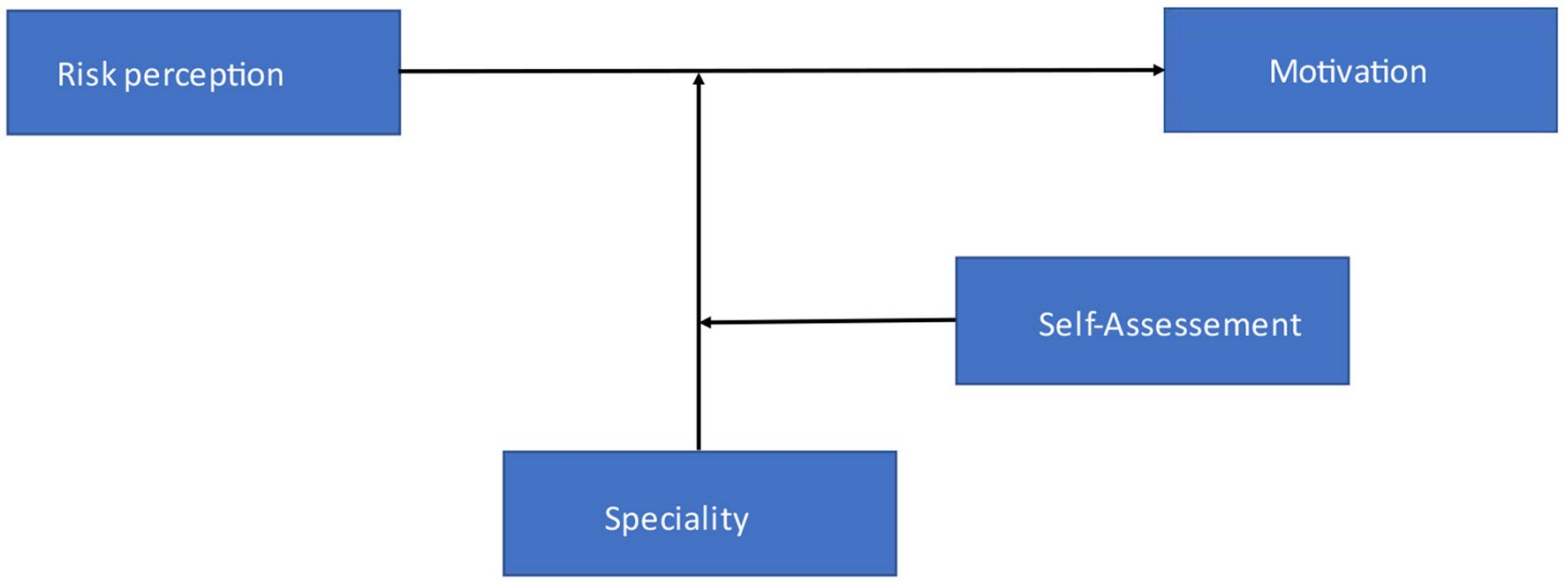
Moderation Model for POCUS for all participants – In this study we examined the impact of risk assessment on learning motivation moderated by the speciality. The moderating effect of the speciality is itself moderated by self-assessment

We used model 3 (see Fig. 1) with two interacting moderators of SPSS Process Macro from Hayes to test the three-way interaction between risk perception, specialty, and self-assessment on learning motivation [42]. Risk perception was included as a predictor, specialty as a categorical moderator (with anesthesiology as a reference category), self-assessment as a continuous moderator and four motivational dimensions as criterion variables in four separate moderation analyses. We used anesthesiologists as the reference (baseline) category for the categorical moderator specialty in our analysis. Anesthesiologists were chosen as they represent the largest specialty group in emergency medicine in Germany and were also the most numerous group in our sample. Using them as the reference point allows us to make clearer comparisons between anesthesiologists and the other specialties—trauma surgeons and internal medicine physicians. This approach helps highlight differences in motivation and self-assessment patterns for POCUS training across specialties, with anesthesiologists serving as a meaningful baseline for interpreting these differences. The model applies the bootstrapping method per default with the deviation correction based on 5000 samples at the 95% confidence interval.

## Results

A total of 132 participants responded (response rate 26.4%), 110 (83.3% of respondents) completed the core part of the survey (without ultrasound loop interpretation), 52 (39.4% of respondents) completed at least two loops, 47 (38.2%) completed the survey with missing items, and 31 (23.4%) completed the entire survey including all items.

Of all participants, 46 (37.4%) were female. The mean age was 36.6 years (SD 7.3 years). 87 (70.8%) participants were anesthesiologists, 10 (8.1%) were internal medicine physicians, 18 (14.6%) were surgeons, and eight (6.5%) were others. Of all respondents, 62 (50.4%) had never attended a POCUS course.

The overall motivation to learn POCUS competencies was high among participants, with particularly strong levels of intrinsic motivation (*M* = 6.1, *SD* = 0.87) and identified regulation (*M* = 6.40, *SD* = 0.68). Extrinsic motivation (*M* = 2.3, *SD* = 1.1) and amotivation (*M* = 1.6, *SD* = 0.8) were notably low. Self-assessment among the sample was moderate (*M* = 3.6, *SD* = 0.6). Despite this, the proportion of correct diagnoses for specific cases involving ultrasound loops was modest, with participants correctly identifying 60% of pathologies on average (*M* = 0.6, *SD* = 0.2), whether they reviewed two cases or any number up to all eight.

For all participants, we found no overplacement effects towards their group (for one’s competencies: mean (*M*) = 3.52, standard deviation (*SD*) = 0.56, and for other physicians’ competencies: *M* = 3.40, *SD* = 0.62, *t* (85) = 1.93, *p* = 0.05). Therefore, hypothesis 1a, the presence of overplacement, was rejected.

The paired t-Test revealed that participants do not rate their competencies higher than their performance on the ultrasound loops (*t* (51) = 0.46, *p* = 0.63). Hence, hypothesis 1b (the presence of overestimation) was also rejected indicating as physicians did not rate their performance regarding POCUS to be higher than it actually is.

Relative self-assessment exhibited a moderate negative correlation with intrinsic motivation (*r* = − 0.41, *p* < 0.001) and identified regulation (*r* = − 0.41, *p* < 0.001), as well as a weak positive correlation with amotivation (*r* = 0.28, *p* = 0.01). This suggests that higher relative self-assessment, indicating overplacement, is associated with lower intrinsic motivation and identified regulation, as well as higher levels of amotivation. No significant correlations were found between external regulation and relative self-assessment. In contrast, the self-evaluation to accuracy ratio was significantly positively correlated with external regulation (*r* = 0.36, *p* = 0.01), indicating a medium effect size. This implies that physicians who have a higher self-evaluation to accuracy ratio (indicating overestimation of their performance) tend to report greater levels of extrinsic motivation. Thus, hypothesis 2 was confirmed.

After performing the two-step cluster analysis with intrinsic motivation, external regulation, and identified regulation (amotivation was excluded due to the strong correlation to identified regulation and intrinsic motivation), the self-assessment of one’s competencies and performance regarding diagnostics based on the ultrasound loops as the continuous variables and the maximal risk as a categorical variable using log-likelihood instant measure and after choosing BIC and AIC Criteria, the analysis showed an optimum of two clusters. However, the Silhouette measure of cohesion and separation was poor (< 0.25). In conclusion, physicians could not be grouped based on overestimation, motivation to learn, and risk perception. Hypothesis 3 was, therefore, also rejected.

### Other analyses

The regression analysis explored how perceived risk, self-assessment of competencies, and medical specialty, along with their interactions, influenced amotivation among physicians. In this analysis, anesthesiologists served as the reference category.

Overall, we found that perceived risk did not have a significant impact on amotivation, as indicated by the non-significant coefficient. When comparing different specialties, trauma surgeons were notably less amotivated than anesthesiologists, as reflected in a significant negative coefficient. However, no significant differences were found between internal medicine physicians and anesthesiologists in terms of amotivation.

We also looked at how these factors interacted. The interaction between perceived risk and internal medicine physicians did not significantly affect amotivation, but the interaction between perceived risk and trauma surgeons was significant, suggesting that the combination of these factors plays a role in reducing amotivation among trauma surgeons.

Self-assessment of competencies alone did not show a strong relationship with amotivation. Additionally, higher-order interactions—such as those involving perceived risk, specialty, and self-assessment— also did not yield significant effects. The exception was the three-way interaction between perceived risk, trauma surgeons, and self-assessment, which was significant. This finding suggests that trauma surgeons who perceive higher risks and have a lower self-assessment profile are more likely to experience amotivation.

In contrast, there were no significant three-way interactions in the associations between risk perception, intrinsic motivation, and identified and external regulation.

**Fig. 2:**
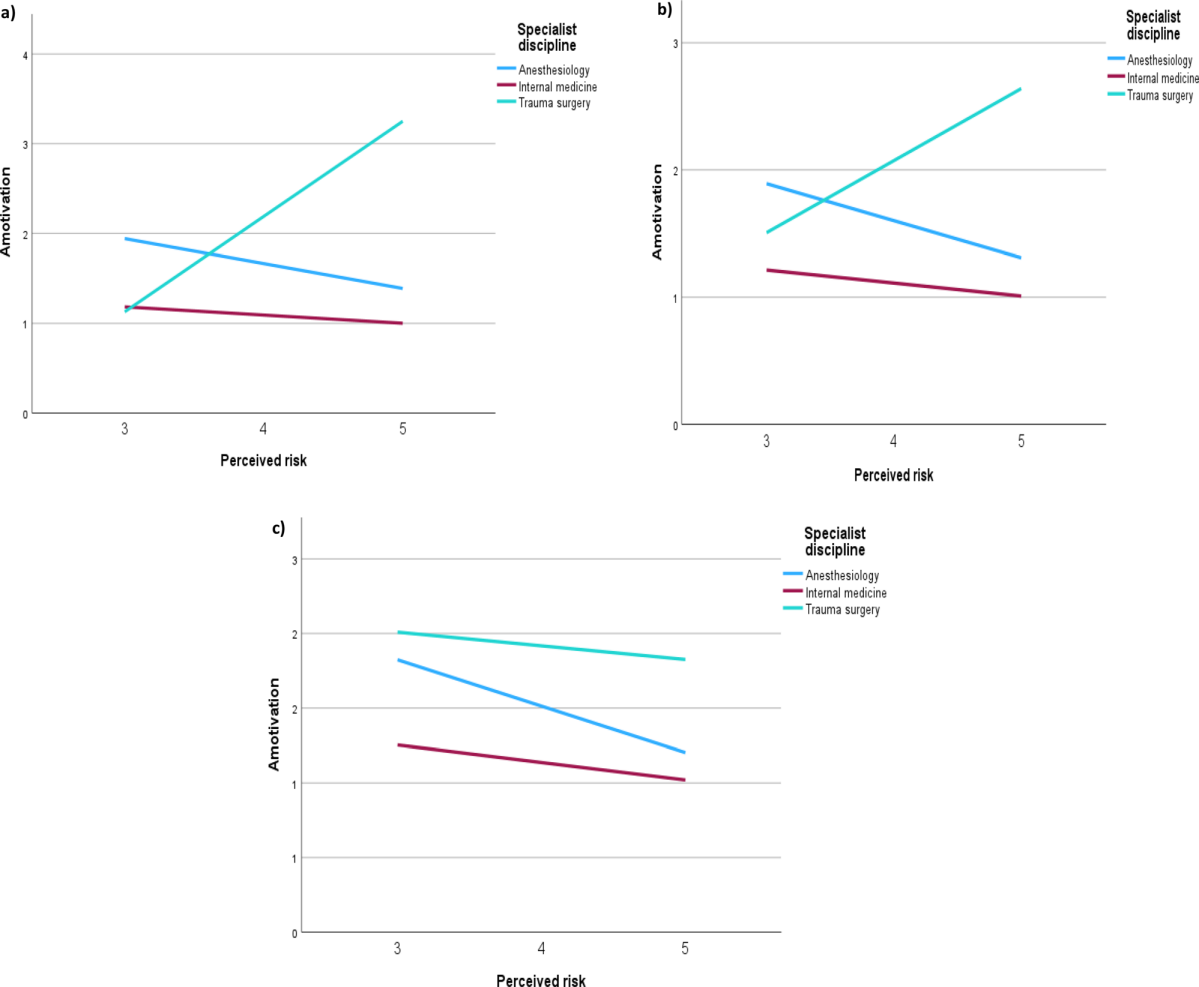
Moderating effect of specialist discipline on the relation between perceived risk and amotivation among physicians: **a**) with lower self-assessment of one’s own competencies (Mean (*M*) = 3 on an ascending Likert scale from 1 to 5) **b**) with moderate self-assessment of one’s own competencies (*M* = 3.5 on an ascending Likert scale from 1 to 5) **c**) with high self-assessment of one’s own competencies (*M* = 4.2 on an ascending Likert scale from 1 to 5) X-axis depicts perceived risk for patients after incorrectly performed POCUS procedure from 3 “medium”, 4 “critical” to 5 “lethal”. Left Y-Axis shows amotivation (1–7 points with 7 as maximum amotivation). The specialist disciplines displayed are anaesthesiology (blue), trauma surgery (green) and internal medicine (red). **(a)** and **(b)** Lower and moderate self-assessment show that anaesthesiologists’ amotivation lowers with higher risk-assessment. In contrast, surgeons’ amotivation rises with higher risk-assessment. There is no significant correlation between perceived risk and amotivation among internal medicine physicians. **(c)** Perceived risk does not correlate with amotivation among surgeons and internal medicine physicians with high self-assessment of one’s own competencies. Among anaesthesiologists the risk-perception is negatively correlated with amotivation

Simple slopes analysis revealed that the higher risk awareness concerning POCUS for anaesthesiologists resulted in lower amotivation, independent of their self-assessment. For surgeons, higher risk stratification for POCUS leads to higher amotivation, especially in the case of poor self-assessment. No interaction was found for internal medicine physicians (see Table 2; Fig. 2a, b and c).

**Table 2:** Analysis of two interacting moderators, specialist discipline and self-assessment of one’s own competencies, on the association between risk-perception and amotivation

Predictor	B	B BCa 95% CI lower	B BCa 95% CI upper
constant	2.81	-3.67	9.29
Perceived risk	-0.19	-1.81	1.43
Internal medicine^1^	-1.73	-24.70	21.25
Trauma surgeons^1^	-16.01	-30.72	-1.30
Interaction_1: Perceived risk*internal medicine^1^	16	-4.82	5.14
Interaction_2: Perceived risk*trauma surgeons^1^	4.21	0.72	7.70
Self-assessment of one’s own competencies	-0.01	-1.90	1,87
Interation_3: Perceived risk* Self-assessment	-0.03	-0.50	0.44
Interaction_4: Self-assessment*internal medicine^1^	0.14	-5.51	5.79
Interation_5: Self-assessment*trauma surgeons^1^	3.73	-0.11	7.57
Interaction_6: Perceived risk*internal medicine^1^ * Self-assessment	0.01	-1.24	1.26
Interaction_7: Perceived risk*trauma surgeon^1^ * Self-assessment	-0.96	-1.88	-0.03

Criterion variable is amotivation ,^1^Referent category is anesthesiologist, B: unstandardized regression coefficient, B BCa 95% CI lower and B BCa 95% CI upper: lower and upper limits of bias-accelerated bootstrapped 95% confidence interval based on 1000 samples

## Discussion

Our study confirmed that the motivation to learn POCUS among emergency medicine physicians is high, particularly intrinsic motivation and identified regulation, which is critical for sustained learning and competency. This is consistent with previous research [43, 44] and highlights the significance of intrinsic motivation for professional development in medical education and POCUS. However, overconfidence effects, as measured by overplacement and overestimation, were not significantly present in our sample. This contrasts with findings in other areas of medical education, where overconfidence is more prevalent, such as hand hygiene [34, 35, 38, 45–47], basic life support [36] and second victim phenomena [38]. This may suggest that, within the domain of POCUS, physicians have a relatively accurate assessment of their abilities, or it could be due to the small sample size that is not representative of the broader population of emergency medicine physicians. However, the results of various studies indicate that physicians do not rate themselves highly regarding POCUS competencies [48, 49] and that they are reasonably accurate in their self-assessments [50].

Interestingly, while overplacement and overestimation were not significantly detected, relative self-assessment was negatively correlated with intrinsic motivation and identified regulation. This suggests that physicians who overplaced their abilities compared to their peers were less intrinsically motivated to learn. On the other hand, self-evaluation to accuracy ratio was positively correlated with external regulation, indicating that those who overestimated their abilities were more likely driven by external factors, such as recognition or rewards. These correlations underscore the complex relationship between self-assessment and motivation in medical education. Alternatively, it is possible that we did not observe ceiling effects in self-assessment for the first time because the physicians perceived this competency as particularly challenging [51]. This may have resulted in a more balanced distribution of individuals who were both under- and overconfident, which could explain the observed correlations. The complexity of the task might have led to more honest self-reflection, capturing the entire range of self-assessment abilities [50, 52].

Our hypothesis that physicians could be grouped into distinct learner categories (H3) was not supported. While previous studies in other medical competencies identified or used clusters of learners [36, 37, 53], our analysis did not reveal such clear distinctions in the POCUS context. This may be due to the unique nature of POCUS training, where the technical and cognitive demands differ from other medical competencies [54], perceived difficulty or the relative novelty of POCUS in Germany. Alternatively, the lack of distinct groups could suggest that the sample was more homogeneous in terms of motivation and competency, or that the measures used were not sensitive enough to detect meaningful differences.

Finally, our post hoc analysis exploring the interaction between risk perception, specialty, and self-assessment of competencies on learning motivation yielded interesting findings. We discovered that trauma surgeons who perceived higher risks associated with POCUS and had lower self-assessments were more likely to experience amotivation. This indicates that the interplay between perceived risk and self-assessment significantly impacts motivation to learn POCUS, especially in high-stakes specialties. Conversely, higher risk awareness among anesthesiologists was associated with lower levels of amotivation, suggesting that risk perception may have a protective effect on motivation in this group. However, it is essential to note that this does not imply that surgeons are unwilling to take risks. In fact, our analysis revealed that, when self-assessment is not taken into account, trauma surgeons generally display lower levels of amotivation compared to anesthesiologists, even in the presence of higher perceived risks. However, when self-assessment is included in the analysis, a distinct pattern emerges: trauma surgeons who lack confidence in their abilities may experience increased amotivation as patient risk rises. In contrast, anesthesiologists demonstrate relatively stable levels of amotivation, regardless of their self-assessment, suggesting that their motivation to learn is less dependent on their confidence in their competencies and more influenced by their perception of risk. Although the groups of anesthesiologists, trauma surgeons, and internal medicine physicians were unequal in size, as noted in our limitations, and our findings are post hoc, they offer first insights of these interactions that may inspire future research. We acknowledge that this imbalance, coupled with convenience sampling, introduces potential statistical bias and limits generalizability to the broader population. To address this, we used bootstrapping to enhance robustness. Despite these limitations, we believe this finding may reflect a real phenomenon worth further investigation. This pattern, though complex, may be linked to established psychological constructs of risk aversion and task avoidance in high-stakes environments, that have been already observed in emergency healthcare settings [55, 56]. Task avoidance often occurs when individuals with low self-efficacy perceive certain tasks, such as POCUS, as risky due to possible misdiagnosis or errors. This perception can lead persons to avoid POCUS or to pass it to those they consider more skilled, despite its potential benefits for patient care [57]. These findings emphasize the need for targeted motivational strategies that consider the unique contexts of each specialty, focusing on balancing risk awareness with support to enhance confidence in learning.

Our study revealed that nearly half of the participating physicians had not received formal training in POCUS, which likely contributed to their modest performance in accurately interpreting the ultrasound loops [58]. There is evidence that training programs can significantly enhance POCUS competencies among physicians [59–61]; however, retention of these skills remains a challenge [10]. Our post hoc analysis, which found no significant differences in diagnostic performance between those with POCUS certification or trainer experience and those without formal training or with a non-certified course, aligns with findings in the literature [10]. Motivation and self-assessment scores were also similar across groups, supporting previous research that motivation alone does not ensure mastery of POCUS skills in the absence of structured and ongoing practice. These results were based on an independent samples t-test with BCa 95% confidence intervals, using 5,000 bootstrap samples. However, our sample size for participants with diagnostic performance data may have limited our ability to detect moderate or smaller differences. Furthermore, the lack of both formal education and consistent hands-on practice limits the development of essential diagnostic skills [58], leaving physicians less equipped to identify critical pathologies. Although participants demonstrated high motivation, especially intrinsic motivation, the absence of structured, ongoing training and insufficient practical supervision could have hindered their mastery of POCUS [62].

It is well-established that only highly competent practitioners can truly leverage POCUS to benefit patient outcomes [9, 63]. The sensitivity and specificity of POCUS in emergency settings vary significantly across studies, with results ranging from very good to poor [64–66]. This variability can largely be attributed to the competency level of the performer [66, 67]. Thus, competency in POCUS is not only desirable but crucial for delivering accurate diagnostics in high-stakes environments. These findings reinforce the importance of robust training programs, coupled with continuous practice, supervision, and feedback, as essential components in acquiring and maintaining the competencies necessary for effective POCUS use [62].

### Limitations

Our study faces several limitations. The first was the small sample size with a high response burden and a large percentage of anaesthesiologists compared to other specialties. Nevertheless, our data was collected from multiple institutions and clinical groups involved in emergency medicine enhancing meaningfulness. However, our non-representative sampling method may have introduced selection bias, limiting the generalizability of our findings to the broader population of anesthesiologists, trauma surgeons, and internal medicine physicians. Response bias is another limitation; out of 500 physicians contacted, only 31 completed all items, including the eight loops. Additionally, we inquired about experiences with a POCUS course, even though half of the participants had not attended any formal POCUS training. Future studies with larger sample sizes should evaluate the role of a POCUS user’s specialty or role in the POCUS-using environment, especially as we found differences for specialties in the moderation and subgroup analyses.

Second, we used videos in a single ultrasound plane and fictional patient vignettes as a surrogate for real cases. However, case vignettes are acknowledged in medical education for robustness and validity [68]. Additionally, some participants accessed the survey via smartphone, resulting in a lower display quality of the loops. As a result, our ultrasound loops, especially when viewed on a smartphone, may be limited in representing actual clinical cases. On the other hand, smartphone-based POCUS with high resolution is starting to play a role in prehospital emergency medicine and might be utilized in clinical practice [69].

Third, generalization to other countries may be limited, as in Germany, anaesthesiologists are also critical care physicians. In many other countries, emergency and critical care medicine are separate specialties. Additionally, POCUS is not an integral part of all specialties and its implementation in clinical practice varies. In recent years, however, the German Association for Ultrasound (DEGUM) has developed programs for POCUS training that are not limited to the specialty and can be attended by all physicians who meet the criteria. In Germany, these programs are mainly attended by internal medicine physicians, traumatologists, radiologists, visceral surgeons, anesthesiologists, general practitioners, and pediatricians. Therefore, researchers in other countries should concentrate on possible differences in those specialties.

Fourth, we computed the proportion of correctly identified pathological conditions only when two ultrasound loops were responded to. Some participants might have omitted various loops since they could not identify the pathology and proceeded with the questionnaire. We did not characterize the non-response as incorrect in such cases. In future studies, this finding should be reevaluated critically.

Fifth, this was a cross-sectional study that cannot be used to infer causality. This refers, particularly, to the results of the moderation analysis. Further, we used the translated SIMS originally developed for physical activities. However, this score showed acceptable factor structure in preceding work in medical education [40] and German translation [35, 36] with the need for ongoing evaluation.

Sixth, the simple slope analysis has some limitations. The varying group sizes among the different specialities could have influenced the results. For example, there were only ten internal medicine participants in the sample. The restrictive variance limits the interpretability of the results of this analysis.

Seventh, the real-life implications of these psychological findings on overconfidence and learning motivation remain unknown. This first investigative study, which was intended to search for the effect, not its consequences, cannot answer this question. However, the mere existence or absence of over- or underconfidence effects does not categorically lead to the conclusion that occurrence of the effects may be harmful or protecting.

### Interpretation

Regarding the limitations and demand for future investigations in larger sample sizes, we conclude that overconfidence effects (H1) and learning patterns (H3) may not be as present in POCUS as in other established fields of medicine. Further, we conclude that learning motivation in POCUS is a complex topic that is not easily assessed and partially dependent on risk assessment, self-assessment and the user’s role in POCUS-adapting environments (H2). Consequently, trainers, medical educators, educational researchers, and curriculum developers should focus on the learners’ homogeneity on the one side and diversity on the other side to develop tailored, efficient, and sustainable training in POCUS for beginners, fellows, and experts. This is crucial, as POCUS is an emerging skill in most medical disciplines and amongst many medical professionals, including paramedics or specialized nurses, which needs to be mastered in order to reduce misconceptions, medical errors, and inefficient learning formats relevant to patient, workplace and institutional safety.

## Electronic supplementary material

Below is the link to the electronic supplementary material.

Supplementary Material 1

## Data Availability

Data is available on request by contacting the corresponding author (Stefan Bushuven MD M.Sc. M.A. DESA EDIC, Institute for Infection Control and Infection Prevention, Virchowstrasse 10 78224 Singen Germany, Email: Stefan.Bushuven@notis-ev.de
