# Supplementary material for: Self-assessment and learning motivation in emergency point-of-care ultrasound: an online pilot investigation in German physicians
Source: BMC Emerg Med. 2024 Dec 18;24:235. doi: 10.1186/s12873-024-01154-z (PMC11656859; doi:10.1186/s12873-024-01154-z)
Supplement: Supplementary file 1 — Supplementary Material 1 [file 12873_2024_1154_MOESM1_ESM.pdf]

SUPPLEMENT 1

Case	Vignette	Assessment goals
1	You care for a 25-year-old male after a motorcycle accident in the emergency department. POCUS shows the video above.<br><br>A: endotracheal tube, etCO 34 mmHg<br>B: ventilated, FiO2 0.7, Bilevel 35/5 mbar, RR 20, SpO2 100%<br>C: NIBP 70/30 mmHg, HR 141/min, ReCap time 5 sec, sinustachycardia<br>D: GCS 3, RASS -5, sedated, Miosis<br>E: Temperature 36,4°C, left forearm fracture, suspected femoral fracture left, multiple bruises<br><br>S: Accident on the highway 45 minutes ago, intubated on site with GCS 6, NIBP 170/100 mmHg, HF 44/min and anisocoria left > right<br>A: not known<br>M: not known<br>P: not known<br>L: not known<br>E: collided with truck (80 km/h) with his motorcycle (at least 80 km/h)<br><br>ABG: pending<br>X-Ray: not conducted yet<br>12 lead ECG: Sinustachycardia, normal axis, no ST changes	**Correct pathology:**<br>Recognition of free perisplenic fluid or suspected rupture of the spleen
2	You care for a 45-year-old female patient with osteosynthesis after an ankle fracture. The nurses called the ICU team when she complained of shortness of breath. POCUS shows the following loops.<br><br>A: patent<br>B: Respiratory distress, RR 25 breaths/min, vesicular breath sounds bilaterally, SpO_2 88%<br>C: NIBP 90/50 mmHg, HR 140/min, ReCap time 2 sec, sinustachycardia<br>D: GCS 15, RASS +2, anxious, moves all extremities, pain in the operated limb.<br>E: temperature 39,1°C<br><br>S: respiratory distress for about 20 minutes, chest pain, dyspnoeic when speaking<br>A: amoxicillin (vomiting)	**Correct pathology:**<br>Recognition of D-Sign

	M: Ibuprofen 400 mg tds, Oxycodone 10mg bd, Enoxaparin 20mg nocte, Levo-Thyroxine 75 µg mane<br>P: history of previous caesarean section, Factor-V-leiden mutation, hypothyroidism<br>L: lunch about 60 minutes ago<br>E: operation due to an ankle fracture 6 days ago, cause of fracture was a sport accident, today she walked a lot, whereafter the pain begun<br><br>ABG: not obtained<br>X-ray: lower leg this morning, good healing process<br>12 lead-ECG: not obtained	
3	You care for an 80-year-old man in the ED with fever and confusion. He has deteriorated in the last few days and had complained of abdominal pain.<br><br>A: patent<br>B: Tachypnoea, vesicular breath sounds bilaterally, SpO2 95 % on room air<br>C: blood pressure 100/60 mmHg, heart rate: 104/min, arrhythmic pulse<br>D: GCS: 13, RASS -1, diffuse abdominal tenderness<br>E: temperature 39.3 °C<br><br>S: generalised abdominal pain of changing intensity for some days<br>A: none<br>M: Warfarin, Ramipril, Amlodipine, Simvastatin, Metoprolol, Citalopram<br>P: atrial fibrillation, coronary artery disease post CABG, depression, peripheral arterial occlusive disease<br>L: for many days nothing except soup and tea<br>E: none<br><br>ABG: K 5,8 mmol/l, Lactate: 4,2 mmol/l<br>X-Ray: not obtained<br>12 lead ECG: atrial fibrillation, HR: 108/min, no signs of ischemia	**Correct pathology:**<br>Recognition of air bubbles in the portal vein, mesenteric ischemia
4	You care for a 52-year-old woman in the ED with severe abdominal pain. The right-sided flank pain started 2 hours ago.<br><br>A: patent<br>B: normal breath sounds, SpO2: 98 % in room air<br>C: blood pressure: 180/110 mmHg, heart rate: 112/min<br>D: GCS: 15, RASS +1, severe right-sided abdominal tenderness<br>E: Temperature: 38,1 °C	**Correct pathology:**<br>normal

	S: severe pain for 2 hours on the right abdominal side<br>A: ACE-inhibitor<br>M: Candesartan, L-Thyroxine, Ibuprofen<br>P: Obesity, hypertension, hypothyroidism<br>L: brunch 2 hours ago<br>E: wedding of her daughter was yesterday<br><br>ABG: normal<br>X-Ray: not obtained<br>12 lead-ECG: sinus rhythm, heart rate 114/min, ventricular extrasystoles about 10/min	
5	A 46-year-old man with left thoracic respiratory pain and mild dyspnoea in the emergency department.<br><br>A: Free<br>B: Vesicular breath sounds bilaterally apically, left basal breath sounds absent, SpO2: 97% in room air.<br>C: RR: 140/80 mmHg, HR: 78/min.<br>D: GCS: 15, left thoracic respiratory pain<br>E: Temperature: 35.8 °C<br><br>S: increasing respiratory and exertional left thoracic pain for days<br>A: none<br>M: sertraline, doxepin<br>P: anxiety disorder, alcohol abuse, nicotine abuse<br>L: sober, copious alcohol consumption last night<br>E: bruising all over the body of varying ages.<br>ABG: pending<br>Radiology: pending<br>12K-EKG: inconspicuous	**Correct pathology:**<br>Hematothorax, pleural effusion
6	You treat a patient in the resuscitation room after she had fallen from a 4m high wall of a ruin. The patient was found unconscious, intubated on site by the first responders and brought to your hospital with the rescue helicopter. Echocardiography shows the above picture.<br><br>A: intubated, bloody secretions orally, stiff neck<br>B: ETT depth 21cm, vesicular breath sounds bilaterally, SpO2: 96 %, FiO2 0.6<br>C: NIBP: 90/50mmHg, HF: 108/min, congested neck veins, soft heart sounds on auscultation<br>D: GCS 3, sedated with propofol and sufentanil.<br>E: temperature: 37.4 °C<br><br>S: unknown<br>A: unknown<br>M: unknown	**Correct pathology:**<br>Pericardial effusion, pericardial tamponade

	P: Scar at the lower creek<br>L: Almost empty snack box in backpack<br>E: Patient is wearing hiking clothes and had a backpack with her<br><br>BGA: not yet performed<br>Radiology: pending<br>12 lead ECG: SR, HF: 114/min, low voltage	
7	You treat a patient in the emergency department and have the indication to insert a CVC. Immediately after the CVC placement, the patient shows a drop in saturation and an decreased breath sounds on the right. You perform chest sonography at various sites and obtain the findings seen in the video in each case.<br><br>A: Clear<br>B: Breath sound decreased on the right, SpO2: 89 %.<br>C: RR: 80/50 mmHg, HR: 142/min<br>D: Patient becomes increasingly anxious and shows motor agitation<br>E: Temperature: 36.4 °C<br><br>S: onset of symptoms immediately after the insertion of the CVC<br>A: Pollen<br>M: Takrolimus, CellCept, ganciclovir, Cotrim, hydrocortisone, folic acid<br>P: lung transplantation 2 years ago for idiopathic pulmonary fibrosis<br>L: Dinner at a restaurant 1 hour ago<br>E: Was discharged from the pulmonary rehabilitation clinic yesterday<br><br>ABG: pO2: 58 mmHg, CO2: 51 mmHg, K 5.0 mmol/l<br>Radiology: CT thorax on CD from the rehabilitation clinic - bronchiolitis obliterans not to be excluded with certainty<br>12 lead ECG: SR, HF: 142/min, questionable ST-segment elevation in V1-V2 (1 mV)	**Correct pathology:**<br>normal
8	You treat a 71-year-old female patient in the emergency department with fever, fatigue, and increasing dyspnoea on exertion. You perform an emergency sonography.<br><br>A: free	**Correct pathology:**<br>Pleural effusion, compression of lung, pneumonia

	B: bilateral crackles from secretions, SpO2: 92 % in room air<br>C: NIBP 130/80 mmHg, HR: 76/min<br>D: GCS: 15, sore throat and fatigue<br>E: temperature 37.9 °C<br><br>S: cold for 2-3 weeks, increasing weakness and fatigue in the last days<br>A: Aspirin<br>M: amlodipine, metoprolol, metamizole, ibuprofen<br>P: hypertension<br>L: Breakfast 3 hours ago<br>E: Cold since 2-3 weeks, in the last days increasing weakness and fatigue<br><br>ABG: pO2: 71 mmHg with 4l oxygen via nasal cannula, CO2: 46 mmHg<br>Radiology: pending<br>12 lead ECG: SR, HF: 68/min, signs of left heart hypertrophy	

Table S1: Detailed fictious patient vignettes shown to the candidates. Each vignette comprises ABCDE for primary, SAMPLER for secondary and further examinations for tertiary survey. Vignettes were presented in German. Supplemental material was translated by the authors into English.

SUPPLEMENT 2

Survey on motivation to learn and medical education in emergency sonography

Dear Sir or Madam,

We would like to invite you to participate in an anonymous survey of about 20 minutes on motivation to learn in the teaching of emergency sonography and thank you already now for your participation.

The background of our project is the recording of motivation hurdles in medical education in the recognition and treatment of sonographically recorded findings from emergency medicine in order to examine current concepts and modify them if necessary.

With this survey we would like to determine in the first part your estimations of your own competences in the field of emergency sonography with the estimation of others.

In the second part, we present some cases and sonography findings for you to evaluate.

With this anonymous (no storage of name or IP address) survey of the Department of Anesthesiology and Intensive Care Medicine of the University Hospital Freiburg, we want to collect these assessments and use them for scientific evaluation.

Our team acts independently from professional societies, market research and industry (no Conflict of Interest).

Your answers will be treated confidentially. The overall results are intended for publication in a medical journal. Participation is voluntary and can be terminated at any time without giving reasons. There are no advantages or disadvantages to participating or discontinuing participation in the survey.

If you have any questions about the survey or the topic, please contact the study directors Dr. Joachim Bansbach and Dr. Stefan Bushuven.

We thank you for your participation!

With collegial regards
Joachim Bansbach, MD & Stefan Bushuven, MD

____

Joachim Bansbach, MD DESA
Specialist in Anesthesiology, ZB Palliative, Intensive and Emergency Medicine
Specialist at the Clinic for Anesthesiology and Intensive Care Medicine of the University Hospital Freiburg
Email: joachim[point]bansbach[at]uni-freiburg.de

Dr. med. Stefan Bushuven MME MA DESA EDIC
Master of Science (Medical Education), Master of Arts (Ethics in Medicine)
Specialist in Anesthesiology, ZB Medical Hygiene, Palliative, Intensive and Emergency Medicine, Clinical Risk Manager ISO 31000
Associate Researcher of the Department of Anesthesiology and Intensive Care Medicine of the University Hospital Freiburg
Email: stefan[dot]bushuven[at]glkn.de

Translated with www.DeepL.com/Translator (free version)

First some questions for you ...

What gender are you? *
female
male
diverse/transgender/transidentical
not specified

How old are you? *
Free-text entry
How old do you feel? *
Free-text entry

To which discipline do you belong? *
Anesthesiology (advanced training)
Anesthesiology (specialist)

Internal medicine (advanced training)
Internal medicine (specialist)
Surgery (advanced training)
Surgery (specialist)
Medical students
not specified

Have you completed training in the following competencies?
Emergency medicine
Intraclinical acute and emergency medicine
Special intensivecare medicine
Special trauma surgery
ATLS
PHTLS
ITLS
TTLS
ACLS
PALS

Do you have any of the following training in emergency sonography ? *
None
Completed course in abdominal/emergency sonography or echocardiography without certificate
Completed course in emergency sonography with certificate
Completed course in echocardiography with certificate
Completed course in abdominal sonography with certificate
Instructor status in emergency sonography
Instructor status in other areas of sonography

What is the best way to describe your work location? *
Non-hospital setting/physician's office
Hospital: basic+ standard care provider (Level 1)
Hospital: Focus provider (Level 2)
Hospital: Central provider / maximum care provider (Level 3)
Hospital: University hospital (Level 3)
Rehabilitation hospital
not applicable

Which device are you using to conduct this survey? *
Desktop/Laptop
Tablet
Smartphone
Other

As a medical professional, have you worked in an intensive care unit or ambulance/emergency department/emergency room in the last 2 years? *
yes
no

TRAINING PARTICIPATION

**If you are attending emergency sonography training, to what extent do the following statements apply to you ? ***<br><br>***Answer options on each question:***<br>strongly agree<br>agree mostly agree<br>somewhat agree<br>moderately agree<br>slightly agree<br>very slightly disagree<br>strongly disagree<br><br>***Items:***<br>I think this training is interesting for me<br>I am doing this training for my own benefit<br>I am doing this training because I have to<br>There may be good reasons to do the training, but I don't see any for myself<br>I think such a training is pleasant<br>I think such a training is good for me<br>I am doing it because it is something I have to do.<br>I do it, but I do not think it is worth the time<br>I do it because it is fun<br>I participate because it is my own choice<br>I participate because I have no other choice<br>I don't know, but I don't see what the training is going to do for me<br>I feel good about myself when I attend the course<br>I think the training is important for me<br>I feel that I have to attend the training<br>I will attend, but I am unsure if it is good to follow something like this
**Self-assessment questions**<br>**I have sufficient knowledge of emergency sonography. ***<br>Strongly agree<br>Fairly true<br>Partly true<br>Somewhat true<br>Strongly disagree<br><br>**I have good motor skills in the use of emergency sonography . ***<br>Strongly agree<br>Fairly true<br>Partly true<br>Somewhat true<br>Strongly disagree<br><br>**I consider emergency sonography to be an essential component in the care of critically ill patients. ***<br>Strongly agree<br>Fairly true<br>Partly true

Somewhat true
Strongly disagree

I am flexible in interpreting emergency sonography when deviating levels of sectioning are required. *

Strongly agree
Fairly true
Partly true
Somewhat true
Strongly disagree

I correct others as appropriate to the situation when I discover errors in the use of sonography. *

Strongly agree
Fairly true
Partly true
Somewhat true
Strongly disagree

I accept situational corrections from others when they correct me on the use of sonography. *

Strongly agree
Fairly true
Partly true
Somewhat true
Strongly disagree

Assessment questions Physicians in further training (anesthesiology).

Have adequate knowledge of emergency sonography. *

Strongly agree
Fairly true
Partly true
Somewhat true
Strongly disagree

Have good motor skills in the use of emergency sonography . *

Strongly agree
Fairly true
Partly true
Somewhat true
Strongly disagree

You consider emergency sonography to be an essential component in the care of critically ill patients. *

Strongly agree
Fairly true
Partly true
Somewhat true
Strongly disagree

They are flexible in interpreting emergency sonography when divergent levels of sectioning are required. *
Strongly agree
Fairly true
Partly true
Somewhat true
Strongly disagree

Correct others as appropriate to the situation when they discover errors in the use of sonography. *
Strongly agree
Fairly true
Partly true
Somewhat true
Strongly disagree

They accept corrections from others as appropriate to the situation when corrected in the use of sonography. *
Strongly agree
Fairly true
Partly true
Somewhat true
Strongly disagree

Assessment questions physicians in training (Trauma surgery)
Same questions as above

Assessment questions Specialists (Anesthesiology)
Same questions as above

Assessment questions Specialists (Trauma surgery)
Same questions as above

Assessment questions senior Anesthesiology residents.
Same questions as above

Assessment questions senior Trauma surgery residents.
Same questions as above

How do you estimate the risk of an incorrectly identified finding in emergency sonography? The credible maximum damage is *
insignificant
minor - but no lasting damage
noticeable- with prolonged stay in hospital
critical - with permanent physical damage
catastrophic - resulting in death

In your setting, how often does it happen that a patient suffers the estimated maximum harm from this? *
less frequently than once in 3 years
more often than once in 3 years
more often than once a year
more often than once in 3 months
more often than once a month

On the following pages you will now see cases and emergency sonographies.<br><br>You can start the videos, unfortunately we cannot offer them as endless loops. Therefore, first read the case vignette and then start the video several times if necessary. Then answer the questions
**Questions on all 8 cases**<br><br>**Please evaluate this emergency sonography in the context of image quality and sound criteria!**<br>Free-text entry<br><br>**How confident are you with the assessment? ***<br>very uncertain - very sure [visual scale 0-100%]<br><br>**Please evaluate this emergency sonography in the medical context of what you see! ***<br>Free-text entry<br><br>**How confident are you with the assessment? ***<br>very uncertain - very sure [visual scale 0-100%]
**Suggestions for improvement, suggestions and comments**<br>**Do you have any suggestions for improvement, special experiences or comments regarding emergency sonography? Let us know!**<br>Free-text entry

Table S2: Questionnaire translated to ENGLISH by the authors. Questions marked (*) were mandatory questions.
